# Effect of the Longitudinal Tensile Creep on the Stiffness of Radiata Pine (*Pinus radiata* D. Don)

**DOI:** 10.3390/ma15124314

**Published:** 2022-06-18

**Authors:** Oswaldo Erazo, Judith Vergara-Figueroa, Paulina Valenzuela, William Gacitúa

**Affiliations:** 1 Department of Wood Engineering, Center for Biomaterials and Nanotechnology, Universidad del Bío Bío, Concepción 4030000, Chile; jvergara@ubiobio.cl (J.V.-F.); nvalenzu@ubiobio.cl (P.V.); 2 Department of Wood Engineering, Faculty of Engineering, Universidad del Bío Bío, Concepción 4030000, Chile

**Keywords:** mechanical properties, modulus of elasticity, radiata pine, longitudinal tensile creep

## Abstract

The influence of load on the cellulose microfibrils of single cells or thin wood foils is known. It can decrease the cellulose microfibril angles and, in turn, increase the stiffness. However, this modification of a piece of wood, which is made up of multiple cells, is unknown. The aim of this research was to study the effect of tensile creep on the longitudinal stiffness of radiata pine wood. The modulus of elasticity of each specimen was determined before and after being subjected to tensile creep. The samples were loaded at 1170 N and 1530 N for 20 min at 70 °C. The load was determined as a function of a percentage of the force at the proportional limit. The moduli of elasticity before and post-tensile creep showed no effect on the stiffness of wood at the macroscopic level, but neither were there damage to the cell structure. It can be assumed that there are changes at the microscopic level, but they are not enough to be reflected at the macro scale. It is also challenging to achieve the modifications that occur at the level of a single cell or in thin wood foils; however, the implications of this would be favorable for the development of stronger wood-based products.

## 1. Introduction

The growing interest in using natural resources to replace materials such as concrete and steel has led researchers to develop new processes for the use of low-carbon materials and products, and their properties meet or exceed the quality and resistance standards established in the regulations. Wood represents a source of raw material that meets all the criteria desired in a material: versatile, ecological, and resistant [1,2]. However, it is an organic material that undergoes a series of physiological processes [3] and its formation is conditioned by genetic and environmental factors [4] that make it an anisotropic material.

As wood is a hierarchically organized composite material, its mechanical properties vary depending on the scale of study [5]. At the microscopic level, it is distinguished that the fibers are made up of three structural polymers, each one with different chemical and mechanical properties, but at the same time, they interrelate with each other, forming a composite. The arrangement of cellulose microfibrils in the wood cell wall and the connection to the hemicelluloses, among others, are also observed [6].

At the macroscopic level, wood density is considered a determining factor in mechanical properties, which varies considerably within the tree [7]. Therefore, it is a very important property for structural designs and experimental tests [8]. Low- and medium-density woods have lower mechanical properties. Radiata pine is a medium-density wood [9] and also has a high proportion of juvenile wood [8]. These factors affect the wood quality. Improving the mechanical performance of this species gives it a competitive advantage over other structurally used timbers.

Numerous treatments have sought to modify wood to improve its properties, such as chemical modification processes [10,11,12,13,14,15], thermal treatments [16,17,18,19,20,21,22], thermohydromechanical treatments [23,24,25], and combinations of these with other processes [26,27,28,29,30,31,32]. Although chemical and thermal treatments increase the resistance to biological degradation and improve the dimensional stability [33,34,35], in the latter, mechanical properties decrease due to the temperatures used [36,37,38,39,40]. However, in the densification processes, there is an increase in surface hardness [41,42,43,44,45]. In addition, it has been reported that both the modulus of elasticity and the modulus of rupture increase with the density [46,47,48,49]. Although these processes can improve certain properties in wood, they do not focus on increasing the stiffness. Any process to modify wood requires an understanding of the complex mechanical behavior of wood, which is a function of several parameters derived from its intrinsic nature and external factors [50]. On the other hand, wood is a viscoelastic material; therefore, its mechanical properties obey the elastic behavior of the cellulose and the viscous behavior of the lignin-hemicellulose matrix. Hemicelluloses act as a mediator between cellulose and lignin by providing flexibility among the cellulose chains through hydrogen bonds, which can easily break and re-form again. In addition, hemicelluloses bind to lignin through covalent bonds [6,51].

Polymeric materials that are subjected to tensile deformation present a progressive alignment of their polymeric chains in the direction of the applied stress [52]. The application of temperature and a constant tensile load over time is known as tensile creep or thermomechanical creep. When wood is subjected to tensile creep, it can undergo deformations. Temperature and humidity conditions make its time-dependent behavior very complex [53]. In the glassy state, the structural components of wood remain completely unchanged; there is no thermal movement [54]. Temperature increases this movement [54,55]. This allows the matrix to soften, changing from a glassy to a rubbery state. Once the temperature is decreased, the matrix will return to its glassy state, preserving the elastic deformations of the microfibrils and the matrix [56].

Additionally, when wood is exposed to external stress, the microfibrils bear much of the load. Increasing stress produces a shearing deformation that makes the microfibrils slide over each other in the lignin-hemicellulose matrix through a stick-slip molecular mechanism-type Velcro connection, resulting in the breakage and continuous formation of hydrogen bonds between fibrils-fibrils and fibrils-matrix [57]. This allows the reorientation of the microfibrils or, to a certain degree, the decrease in the microfibril angles [56,58]. Much research on the mechanical characterization of individual fibers has been compiled in a review, in which the deformation mechanisms experienced by fibers exposed to stress are described [5]. Some degree of alignment of the cellulose chains of herbaceous plant fibers, subjected to cycling loads and subsequently to a monotonic stress test, has also been reported. This alignment produced a slight increase in the modulus [59]. Similar behavior has been found in other herbaceous fibers subjected to different load cycles [60,61,62]. On the other hand, the tensile mechanical behavior of wood sheets (thickness in microns) has also been studied [57,63,64,65], as well as the influence of the microfibril angle [66] and the moisture content in very thin wood tissues subjected to longitudinal tensile creep [67]. Kamiyama et al. investigated the effect of tensile loading on the angle of microfibrils in compression wood tissues, finding that they tend to align [68] and decrease the microfibril angle in the axial direction to the cell axis and load [69]. This mechanical behavior establishes a relationship between the elongation and the decrease in the microfibrils angle [64]. Therefore, this mechanical response of wood turns out to be an advantage in species that have little structural stiffness. Until now, the influence of the load on the microfibrils of a single cell or thin wood foils is known, which can cause a decrease in the cellulose microfibril angles and, in turn, increase the stiffness. However, considering the mechanical behavior of wood as a function of its anisotropy, the question arises: can the same phenomena occur in a piece of wood composed of multiple cells? This research aimed to study the effect of tensile creep or tensile longitudinal deformation on the longitudinal stiffness of radiata pine wood.

## 2. Materials and Methods

### 2.1. Sample Preparation and Conditions

Strips with dimensions 50 × 50 × 1600 mm in the tangential, radial, and longitudinal directions, respectively, were obtained from a 26-year-old radiata pine (*Pinus radiata* D. Don) tree from plantations, located on the San Luis property of the company Aserraderos JCE S.A., Los Angeles, Bío Bío Province, VIII Region, Chile. The tree was sectioned from the base up to 4 m. The section from 1.5 m was used. The wood was dried to 12% moisture content in an industrial kiln. The strips were obtained from a single tree to reduce the wood variability [70]. The position of the wood piece from the pith to the bark was not considered in this research, as there is a high anatomical variability between these two zones. Based on the influence of density on wood mechanical properties, the density of radiata pine was considered. This way, all the strips located from the pith to the bark were taken to find the basic density. The basic density of the strips was determined according to the ASTM D2395-14 standard [71]. From the density results obtained, two densities 0.39 and 0.43 g cm^−3^ were selected to determine the effect on the response variables.

**Figure 1:**
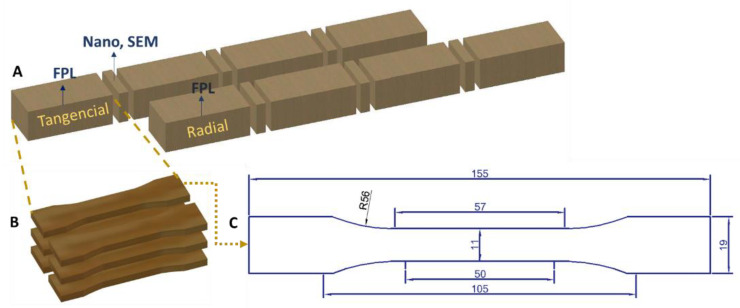
Diagram for obtaining the specimens in a strip: (**A**) sections in a wooden strip: tangential and radial; (**B**) dog bone specimens per section; (**C**) dimensions in millimeters (mm) of the dog bone specimen.

Each strip was divided into two sections. The first section was cut in the tangential direction and the second part was cut in the radial direction. From each section, 4 subsections of 6 specimens each were obtained. The first set of specimens was used to determine the force at the proportional limit, and the following subsections were subjected to treatment. In addition, small blocks of wood of 30 mm^2^ were considered as control samples for the mechanical tests and analysis by electron microscopy, SEM (Figure 1).

**Figure 2:**
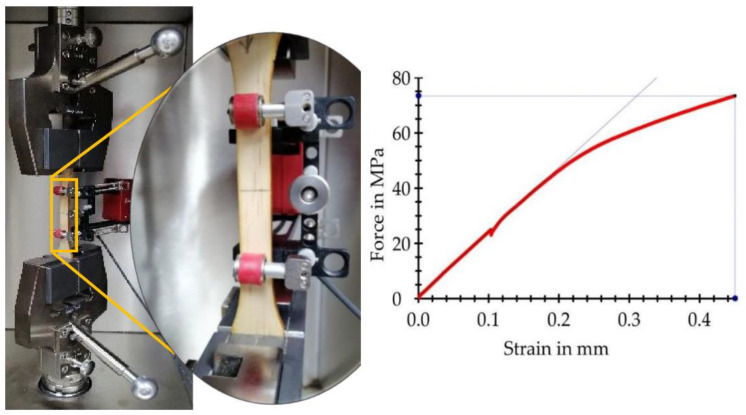
Clip-on extensometer. Determination of the force at the proportional limit.

Modified dog bone specimens were made according to the ASTM D638-14 standard [72]. A laser cutting machine was used to obtain homogeneous sample edges. The specimen design considered the area of longitudinal variation between two points of the clip-on extensometer (Figure 2). Samples free of knots, cracks, or any other defects were used for the tests. They were also conditioned in a climatic chamber (Memmert, Schwabach, Germany) to an equilibrium moisture content of 12% (temperature = 20 °C and relative humidity = 65%).

### 2.2. Load Determination for the Tensile Creep Test

Specimens from each strip (Figure 1) were subjected to a longitudinal tensile test in a Zwick/Roell universal machine (Zwicki Z5.0 TN, Ulm, Germany) equipped with a load cell of 5 kN. The test speed was 5 mm min^−1^. The load used during the tensile creep test was determined at the proportional limit of the pieces, that is, at the point where the proportionality line diverges from the stress–strain curve (Figure 2). The force at the proportional limit of the specimens with both the radial and tangential cuts was calculated using Equation (1):

(1) $$F_{PL}=\sigma_{PL}\times A$$

where $F_{PL}$ is the force at the proportional limit, $\sigma_{PL}$ is the stress at the proportional limit, and *A* is the cross-sectional area of the specimen.

In tests prior to this research, it was found that some pieces subjected to the force at the proportional limit value or higher presented partial or total fractures. For this reason, 65% and 85% of the load at the proportional limit, that is, 1170 N and 1530 N, respectively, were established as conditions for the applied treatments. These percentages of the force at the proportional limit are very similar to those used in other investigations [63,66].

### 2.3. Determination of the Modulus of Elasticity of Specimens before and after the Creep Tests

The modulus of elasticity of each specimen was determined before and after the tensile creep test. This allows the determination of the effect of the tensile creep on the stiffness of wood. A clip-on extensometer was used to determine the modulus of elasticity in the deformation range between 0.05 and 0.20%. In this range, no damage to the specimen was caused, as it was below the proportional limit.

### 2.4. Tensile Longitudinal Creep Test

**Figure 3:**
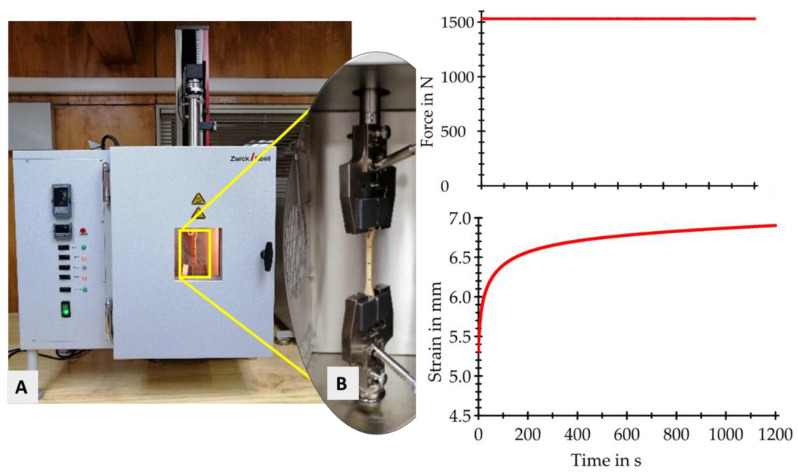
Tensile creep test: (**A**) testing machine equipped with temperature chamber; (**B**) specimen during tensile creep test.

The creep process was performed in a Zwick/Roell testing machine (Zwicki Z5.0 TN, Ulm, Germany), coupled with a Zwick/Roell heating chamber (Figure 3). Before the creep test, the samples were conditioned in a climatic chamber with a temperature of 70 °C and relative humidity of 78%. The temperature used was equivalent to that of the thermal chamber. The specimens were wrapped in transparent plastic paper to prevent moisture loss during the creep test. The loads applied to the specimens were 65% and 85% of the force at the proportional limit. The loading rate was 50 N min^−1^. After reaching the creep load, the specimens were loaded for 20 min at 70 °C. After that time, the specimens were unloaded at a rate of 1 mm min^−1^ to avoid damage. Afterward, the post-creep modulus of elasticity for each specimen was determined.

### 2.5. Morphological Changes of the Samples Subjected to Tensile Creep

Control specimens were taken as described in Section 2.1, while for the treated samples, a small portion of the narrow section of the dog bone specimen was studied. Untreated and treated samples were vacuum-dried for 24 h at 70 °C. They were then placed in a Denton Vacuum sputter metallizer to be coated with gold for 30 s. The small blocks of wood were studied using a scanning electron microscope (SEM), JEOL JSM-6610LV (JEOL, Tokyo, Japan). Morphological changes and the presence of microcracks in the wood cell wall were evaluated. Wood cell images were taken at 10 to 15 kV with a magnification up to 500 times.

### 2.6. Statistical Analysis

An analysis of variance was performed using the statistical program SPSS version 21 (IBM Corp., Armonk, NY, USA). The significant difference was determined with *p* < 0.05 for all the conditions evaluated.

## 3. Results and Discussion

### 3.1. Modulus of Elasticity of the Specimens before Tensile Creep

Table 1 shows the average values of the moduli of elasticity obtained for specimens with densities 0.39 and 0.43 g cm^−3^. The moduli of elasticity found in this research are similar to those obtained by some researchers [73,74]. However, there are reports in the literature of moduli with lower values [75,76].

The mean moduli of the specimens for each condition are shown above. As indicated in the methodology, the modulus of elasticity of each specimen before being subjected to creep was determined between 0.05% and 0.2% deformation percentages. The same piece was then tested again after being exposed to the creep process. This way of evaluating contributes to high accuracy in detecting potential changes, at the macroscopic level, in the wood stiffness, as it reduces the effect of heterogeneity if it had evaluated paired specimens. This was studied in a previous test, evaluating paired specimens, i.e., side by side, with an apparent minimal anatomical variability. In this regard, one specimen was used as a control and the other one was subjected to the treatment. The control and treated specimens showed different moduli and a high variability between the different pairs of specimens, generating uncertainty and difficulty in determining if the treatment produced an effect. Consequently, other tests were carried out on untreated paired specimens, obtaining the same tendency as explained above. This behavior is attributed to the high variability found in wood at the macroscopic, microscopic, and ultrastructural levels [77], which is also reflected locally in the mechanical properties.

The highest moduli of elasticity were found in the samples with a density of 0.43 g cm^−3^. Analysis of variance indicated that there were significant differences between the two densities (*p* < 0.05). It is to be expected that even though density is not the main factor that determines mechanical properties [8,78], it is a reference, as the higher the density, the higher the mechanical properties [79]. Regardless of whether the wood had the same density, a variation in the moduli of elasticity can be found, due to the variability in the microfibril angles in the cell wall [78]. Likewise, there is a strong negative correlation between the microfibril angle and basic density, as reported in a study carried out by Yin and collaborators in *Cunninghamia lanceolata* [80]. Additionally, density may vary within a piece because of the presence of both earlywood and latewood. Li and coworkers evaluated wood samples employing dynamic mechanical analysis (DMA). They found a high correlation between density and elasticity modulus of both latewood and earlywood, although in the latter, it was 19% much lower [79]. All the above may explain the values of the variation coefficients of this research, which could have been higher if pieces of different origins or parts of the tree had been used. On the other hand, it has been reported that the mechanical properties in the radial direction are higher than those in the tangential direction [81] due to the influence of the cell geometry in the radial-tangential plane and the rays acting as support lines or bars, preventing layers of different stiffness from slipping [82]. However, in this research, it was found that the tangential pieces with a density of 0.39 g cm^−3^ resulted in higher moduli (≈11 GPa) than the radial specimens (≈9 GPa) of the same density. This mechanical response has also been found in coniferous and broadleaf species [83], in which the tangential pieces reached 17% higher than the specimens tested in the radial direction [84].

On the other hand, for the specimens with the highest density of 0.43 g cm^−3^, both the moduli of the tangential and radial pieces presented relatively similar values. According to ANOVA, there were no significant differences between the radial and tangential cutting planes.

### 3.2. Behavior of the Specimens Subjected to the Tensile Creep

**Figure 4:**
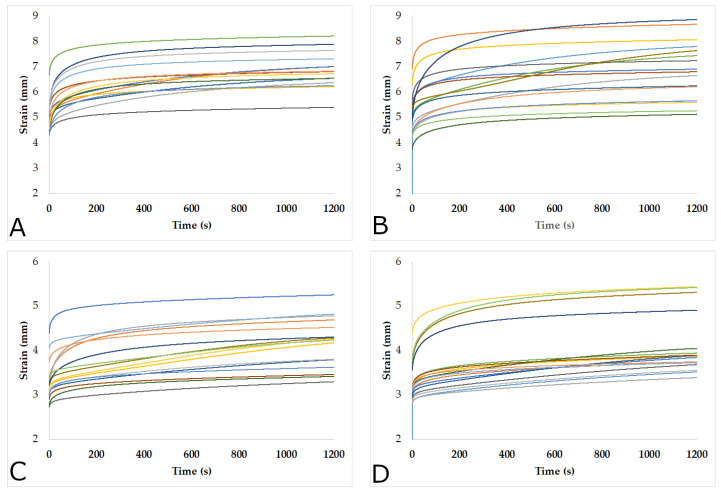
Evolution of the pieces with a density of 0.39 g cm^−3^ subjected to tensile creep: Load 1530 N: (**A**) tangential; (**B**) radial. Load 1170 N: (**C**) tangential; (**D**) radial.

**Figure 5:**
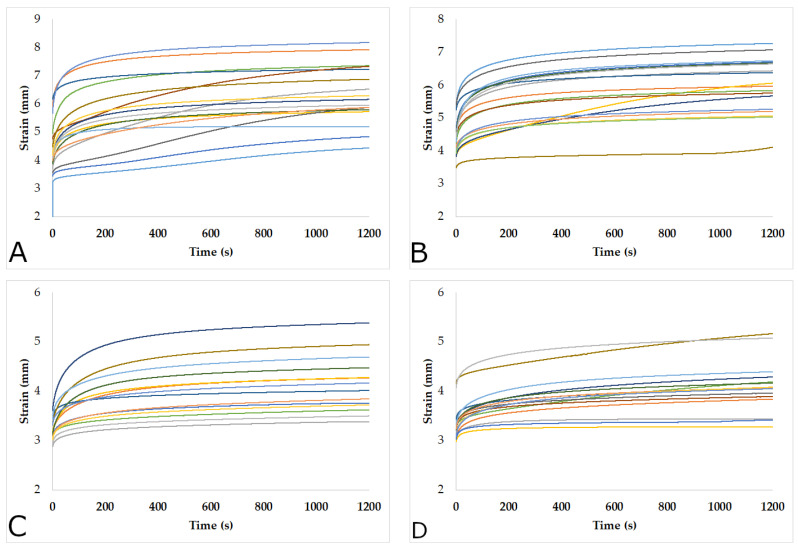
Evolution of the pieces with a density of 0.43 g cm^−3^ subjected to tensile creep: Load 1530 N: (**A**) tangential; (**B**) radial. Load 1170 N: (**C**) tangential; (**D**) radial.

Figure 4 and Figure 5 show the behavior of the specimens with densities of 0.39 g cm^−3^ and 0.43 g cm^−3^, respectively, subjected to tensile creep. Each of the curves represents a specimen. The elongation was obtained by the displacement of the crosshead of the Zwicki machine. It is evident that the specimens had different responses, similar to the variation found in the post-creep moduli of elasticity. In almost all samples, a very rapid increase in strain was observed during the first 100 s, and this continued to increase at a slower strain rate. However, in some pieces, this increase was maintained until the end of the process. These variations were due to the nonhomogeneous distribution of the loads caused by the difference and proportion of earlywood and latewood of the pieces, as well as the differences in the tilt angle of cellulose fibrils in both types of wood [85,86], as the larger the microfibril angles, the greater the increase in the longitudinal creep [66,67,87].

On the other hand, no differences in the deformations of the specimens with different densities and cutting planes were observed. However, as for the loads, the higher the loads, the greater the deformations. Thus, the pieces with both densities (0.39 g cm^−3^ and 0.43 g cm^−3^) and both types of tangential and radial cuts, subjected to the load of 1530 N, showed around 50% greater deformation concerning the pieces loaded at 1170 N. However, the radial specimens loaded at 1530 N tended to deform less than tangential samples. In addition, Figure 4 and Figure 5 show that the different groups of specimens presented a high variability of deformations. This variability in the deformation of the pieces subjected to tensile creep is highly dependent on the microfibril angle [66], which varies in earlywood and latewood [85,86,88].

### 3.3. Moduli of Elasticity in Specimens Subjected to Tensile Creep

Table 1 presents the moduli for all the conditions studied. As with the moduli values reported as controls (before tensile creep), analysis of variance showed that there were significant differences in both the density and in the tangential and radial planes (*p* < 0.05) of samples of 0.39 g cm^−3^, but there was no difference in the cutting planes of specimens of 0.43 g cm^−3^. Likewise, analysis of variance revealed no significant differences for the specimens subjected to the two loads. On the other hand, analyzing the effect of the tensile creep process, it was found that there were no significant differences between the before and post-creep moduli. The modulus values were similar. The minimal variations found may be due to the repeatability error margin of the testing machine. Although the loads produced a greater effect on the deformation of the specimens, there was no evidence of a change in the stiffness of wood; practically, the mechanical properties of wood did not change macroscopically.

As reported in the literature, when wood is exposed to a tensile load, cellulose is responsible for the properties in the longitudinal direction, both strength and stiffness [89,90]; thus, the cellulose microfibrils are the ones that resist the load. The greater the load, the greater the displacement between the microfibrils [57]. The influence of the matrix on the strength is minimal, but it can greatly favor the shear stress between the matrix and the cellulose, especially if the microfibril angle is high [91]. Therefore, a reorientation of the microfibrils occurs, which causes a decrease in the microfibril angles [64]. In this sense, for microfibril displacement to occur, a shearing deformation must be produced, which is caused by external forces. Although the deformations at the molecular level were not evaluated, it is evident from the deformation graphs that the pieces underwent some type of deformation due to the different applied forces of 1170 N and 1530 N.

As indicated in the introduction, the aim of this study was to evaluate whether the changes that occur at the level of a single cell or thin sections of wood, which lead to improved mechanical properties, can also, to a certain degree, increase the wood stiffness at the macroscopic level. Among the factors that could affect the increase in stiffness are the wood structure, which, on the one hand, unlike a cell or thin tissues, is completely rigid, that is, the fibers are cemented. This makes the wood behavior more complex as the deformations can originate within the cell and among cells. On the other hand, wood is composed of earlywood and latewood, where, in this study, it was determined that the pieces mostly had earlywood (>70%). According to Li et al. [79], the presence of both types of wood in a piece influences the mechanical response as they behave differently due to their viscoelastic properties. In addition, this variability is also reflected in the variation in the microfibril angles [92], which is the main factor determining the mechanical properties [93]. Investigations using advanced spectroscopy techniques have reported that for wood having high microfibril angles subjected to tensile stress, the deformations are produced by polymer reorientation and sliding, while for wood with low microfibril angles, stretching of the disordered polymers is generated [65]. This implies that depending on the wood cell wall microfibril angles, the mechanical response, at the macroscopic level, will be greater or less depending on the case. On the other hand, although wood has a viscous behavior in the range of 70 °C and 150 °C due to the lignin-hemicellulose matrix [70], the temperature and wood moisture content applied were not enough to allow the softening of the polymers that make up this matrix. A higher temperature was not considered in this first phase, due to the excessive loss of water from wood and the possible damage to the wood structure because of the application of the loads over time.

### 3.4. Morphology of Wood Cells Subjected to Tensile Stress

**Figure 6:**
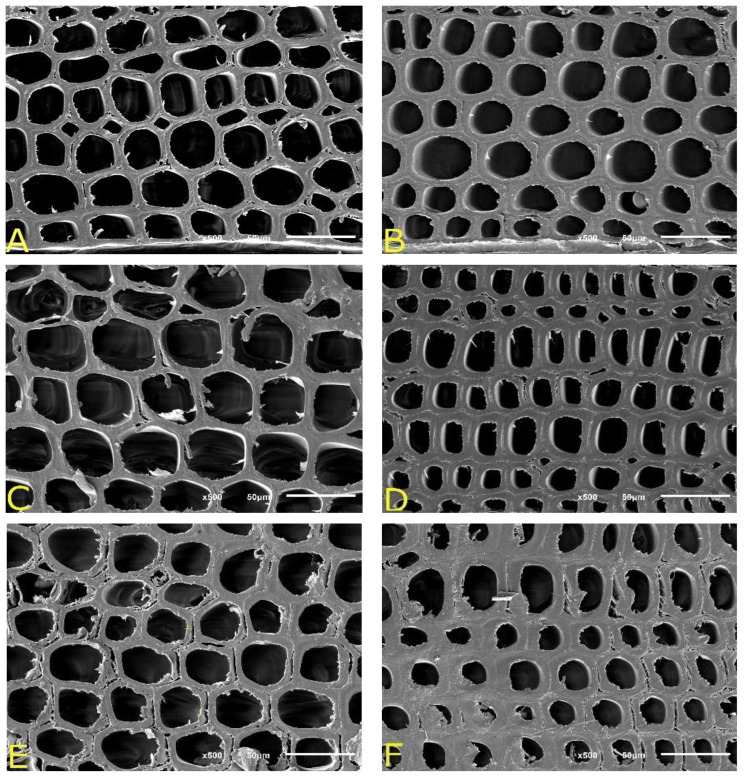
SEM images of radiata pine wood samples: untreated (**A**) earlywood (EW); (**B**) latewood (LW). Samples subjected to tensile creep: (**C**) EW section of a piece loaded 1170 N; (**D**) LW section of a piece loaded 1170 N; (**E**) EW section of a piece loaded 1530 N; (**F**) LW section of a piece loaded 1530 N.

To determine the possible morphological changes experienced by the wood cells, six specimens were selected for each density, as well as the applied loads. The treated pieces were compared with the control samples. The control units were taken according to Figure 1, which were in the same direction and position as the treated ones, that is, each untreated sample was analyzed in parallel with the treated piece. This decreased the effect of wood variability, as discussed above. Regarding the pieces subjected to tensile creep, small blocks of wood were extracted from the middle section of the dog bone specimens. Observations were made on the cross-section. Special attention was paid to the structure of woods having different densities. When analyzing the anatomical structure of control specimens, they showed similar anatomical features (especially fiber size and shape). However, concerning the type of wood (Figure 6A,B), earlywood cells presented on average a lower thickness (3.2 μm) compared to latewood (6.5 μm). Furthermore, it was observed that most tracheids were not hexagonal but tended to be rounded. This geometric form was found in both earlywood and latewood. Additionally, in the earlywood cross-section (Figure 6A), small cracks were observed between adjacent cells, which were the result of stress release during the drying process. Figure 6C,D correspond to specimens subjected to tensile creep at 1170 N. It was observed that the fibers were unaltered, even in both types of wood. There was no evidence that the applied load caused the cells to lose their original shape, as they remained unchanged; however, some samples showed a complete separation of cell walls (Figure 6E,F). This happened in both types of wood, but in the earlywood, tissues were much more severe. It is important to point out that the creep process did not cause these detachments (also present in control specimens), but it can contribute to increasing them due to the applied load and the temperature exposure time. Despite this, there was no decrease in the modulus of elasticity.

## 4. Conclusions

Moduli of elasticity were higher in the specimens having a density of 0.43 g cm^−3^. In this density, the tangential and radial samples presented mean modulus values very close to each other. However, quite the opposite, it was found that in specimens having a lower density of 0.39 g cm^−3^, the behavior of the moduli was very different; the highest values were found in the tangential specimens. The deformations caused by the loads of 1170 N and 1530 N, determined as a function of a percentage of the force at the proportional limit, showed that the higher the load, the greater the deformation, but this had an effect on neither the density nor the wood cutting planes. When comparing the before and post-creep moduli, it became evident that there was no effect of the tensile creep on the wood stiffness at the macroscopic level, but neither were there damage to the cell structure. Based on the applied loads and the temperature, it can be assumed that there were changes at the microscopic level, but they were not enough to be reflected at the macro scale. This research was the basis for the application of a tensile creep process with temperature and humidity conditions higher than those used here. It was also challenging to achieve the modifications that occurred at the level of a single cell or in thin wood foils. The implications of this would be very favorable for the development of better wood-based products.

## Figures and Tables

**Table 1:** Moduli of elasticity of the specimens before (control) and post-tensile creep.

		Basic Density			
		0.39 g cm^−3^		0.43 g cm^−3^	
Cutting Planes	Load	Modulus of Elasticity (GPa)	CV (%)	Modulus of Elasticity (GPa)	CV (%)
Tangential	Control	10.93 ± 1.91 ^a^	17.52	11.06 ± 2.12 ^c^	19.13
	Load 1530 N	10.95 ± 1.97 ^a^	17.99	10.94 ± 1.99 ^c^	18.22
	Control	10.87 ± 1.67 ^a^	15.33	11.40 ± 2.61 ^c^	22.87
	Load 1170 N	10.74 ± 1.66 ^a^	15.47	11.27 ± 2.47 ^c^	21.92
Radial	Control	8.48 ± 1.94 ^b^	22.91	11.21 ± 1.35 ^c^	12.08
	Load 1530 N	8.54 ± 1.87 ^b^	21.90	11.07 ± 1.31 ^c^	11.86
	Control	8.82 ± 1.94 ^b^	22.03	11.36 ± 1.31 ^c^	11.50
	Load 1170 N	8.76 ± 1.92 ^b^	21.88	11.29 ± 1.27 ^c^	11.26

Values are presented as means ± standard deviation. Values followed by different superscript letters in the same column indicate significant differences (*p* < 0.05). CV% = coefficient of variation.

## Data Availability

The data presented in this article are available upon reasonable request from the corresponding authors.
